# Design, Fabrication, and Implementation of an Array-Type MEMS Piezoresistive Intelligent Pressure Sensor System

**DOI:** 10.3390/mi9030104

**Published:** 2018-02-28

**Authors:** Jiahong Zhang, Jianxiang Chen, Min Li, Yixian Ge, Tingting Wang, Peng Shan, Xiaoli Mao

**Affiliations:** 1 Jiangsu Key Laboratory of Meteorological Observation and Information Processing, Nanjing University of Information Science and Technology, Nanjing 210044, China; geyixian@nuist.edu.cn (Y.G.); wangtingting@nuist.edu.cn (T.W.); maoxiaoli@nuist.edu.cn (X.M.); 2 Jiangsu Collaborative Innovation Center on Atmospheric Environment and Equipment Technology, Nanjing University of Information Science and Technology, Nanjing 210044, China; 3 School of Electronic and Information Engineering, Nanjing University of Information Science and Technology, Nanjing 210044, China; chenjianxiang@nuist.edu.cn (J.C.); shanpeng@nuist.edu.cn (P.S.)

**Keywords:** MEMS pressure sensor, array-type, intelligent sensor system, genetic algorithm wavelet neural network (GA-WNN), sensitivity, nonlinear error, temperature drift, hysteresis compensation

## Abstract

To meet the radiosonde requirement of high sensitivity and linearity, this study designs and implements a monolithically integrated array-type piezoresistive intelligent pressure sensor system which is made up of two groups of four pressure sensors with the pressure range of 0–50 kPa and 0–100 kPa respectively. First, theoretical models and ANSYS (version 14.5, Canonsburg, PA, USA) finite element method (FEM) are adopted to optimize the parameters of array sensor structure. Combing with FEM stress distribution results, the size and material characteristics of the array-type sensor are determined according to the analysis of the sensitivity and the ratio of signal to noise (SNR). Based on the optimized parameters, the manufacture and packaging of array-type sensor chips are then realized by using the standard complementary metal-oxide-semiconductor (CMOS) and microelectromechanical system (MEMS) process. Furthermore, an intelligent acquisition and processing system for pressure and temperature signals is achieved. The S3C2440A microprocessor (Samsung, Seoul, Korea) is regarded as the core part which can be applied to collect and process data. In particular, digital signal storage, display and transmission are realized by the application of a graphical user interface (GUI) written in QT/E. Besides, for the sake of compensating the temperature drift and nonlinear error, the data fusion technique is proposed based on a wavelet neural network improved by genetic algorithm (GA-WNN) for average measuring signal. The GA-WNN model is implemented in hardware by using a S3C2440A microprocessor. Finally, the results of calibration and test experiments achieved with the temperature ranges from −20 to 20 °C show that: (1) the nonlinear error and the sensitivity of the array-type pressure sensor are 8330 × 10^−4^ and 0.052 mV/V/kPa in the range of 0–50 kPa, respectively; (2) the nonlinear error and the sensitivity are 8129 × 10^−4^ and 0.020 mV/V/kPa in the range of 50–100 kPa, respectively; (3) the overall error of the intelligent pressure sensor system is maintained at ±0.252% within the hybrid composite range (0–100 kPa). The involved results indicate that the developed array-type composite pressure sensor has good performance, which can provide a useful reference for the development of multi-range MEMS piezoresistive pressure sensor.

## 1. Introduction

To date, microelectromechanical system (MEMS) silicon piezoresistive pressure sensors have been used in a diverse range of commercial and engineering applications including consumer, automobiles, process control, biomedicine, military, meteorology, and aerospace industry areas, and their measurement range and accuracy have been greatly expanded and improved [1,2,3,4,5,6]. The rapid development of market demand puts forward higher requirements for the performance of MEMS piezoresistive pressure sensor. For example, sounding measurements require MEMS pressure sensor not only to measure the high pressure at low altitude accurately but also to measure the low pressure in the airspace sensibly. However, there is always a trade-off between sensitivity and linearity in piezoresistive pressure sensors, and the problem of not getting both high sensitivity and linearity is still not well resolved in the wide pressure range [7,8,9,10]. Thus, the current radiosonde which uses a single pressure sensor usually has different measurement accuracy in two ranges such as 0–50 kPa and 50–100 kPa. The reason is as follows: To meet the very-low-pressure measurement requirement, the load-deflection response of the MEMS pressure sensor is generally maximized by increasing the width and thickness ratio of the diaphragm to achieve higher sensitivity [8,9]. In the case, the non-linearity of the pressure-sensitive film under high pressure is significantly increased and the stability and accuracy of the sensor is compromised [10]. This means the small-range pressure sensor has high sensitivity at low pressure but a severe nonlinear error in measuring high pressure, and even being damaged due to overload. In contrast, the wide-range pressure sensor has the diaphragm with a small aspect ratio and therefore high linearity but low sensitivity when measuring low pressure and easy to produce large measurement error [8,9,10]. To overcome the problem, many design principles and considerations as well as optimization methods have been proposed recently [11,12,13,14,15,16,17], yet a single-range piezoresistive pressure sensor basically has some inevitable shortcomings. For instance, Li et al. [14] proposed a novel structural piezoresistive pressure sensor with four-beams-structured membrane which has achieved a high sensitivity of 25.48 mV/kPa and a low nonlinearity error of 0.75% at full scale (FS), but its pressure range is less than 5 kPa. Rajavelu et al. [15] designed a highly sensitive pressure sensor with excellent linearity extended over 0–54 kPa by employing a 7 μm-thick diaphragm with double Wheatstone bridges, but the work is the only theoretical prediction. Lou et al. [16] presented a nanoelectromechanical system (NEMS)-based piezoresistive pressure sensor by utilizing the SiNWs as the sensing elements, which has relatively high sensitivity of about 0.6% psi^−1^, while Tian and co-workers [17] reported a graphene-based resistive pressure sensor with record-high sensitivity in a wide pressure range (0–100 kPa), but both have low linearity. Thus, it is challenging to use a single wide-range sensor to overcome the difficulty of compatibility between high sensitivity and high linearity.

The array-type multi-range pressure measurement structure opens up a new idea and way to solve the above problem. The array-type pressure sensor system comprises several pressure sensors with different ranges; therefore, it can achieve high linearity and sensitivity within the hybrid composite range and to meet the accurate radiosonde pressure measurement in the full range from 0 to 100 kPa. At present, there have been studies about pressure sensor array [5,9,18,19,20,21,22,23,24]. For instance, Sugiyama et al. [18] reported a 32 × 32 (1k)-element silicon pressure sensor array consisting of piezoresistive elements and CMOS processing circuit, and the array can provide two-dimensional pressure distribution tests for tactile image detection. Kumar et al. [9] fabricated four pressure sensors with different diaphragm sizes simultaneously by putting the different designs on the same mask set so that the best design can be determined after characterization. The different sensors can be used in the specified pressure range for suitable applications. Berns et al. [19,20] proposed a “AeroMEMS” pressure sensor array consisting of 13 individual pressure sensors for high-resolution wall pressure measurements in turbulent flows. Specifically, three different types of sensors (different sensitivities and ranges), featuring a diaphragm thickness of 3 μm and diaphragm sizes of 500 μm, 700 μm, and 900 μm, have been fabricated and attached to the printed circuit board (PCB) with a spacing of 6 mm to form the array, which is mounted flush with the measurement surface. Xiong et al. [21] designed a composite-range micro accelerometer array consisting of four piezoresistive micro accelerometers with the range of 100 g, 500 g, 1000 g, and 2000 g, respectively. The composite-range micro accelerometer has a good linearity and the problem that the acceleration may have a relatively large dynamic range in the same physical process is solved, thus it can accurately measure the acceleration values that cover the high and low range. In addition to optimizing dimensions to enhance the performance of sensor, there are several studies which enhanced the resolution and sensitivity of the piezoresistive sensor array by changing the material [22,23,24], for example, Kottapalli et al. [23] developed a MEMS array of ten pressure sensors using flexible liquid crystal polymer (LCP) as the sensing membrane material for fish-like underwater sensing. The sensor array can passively sense underwater objects by transducing the weak pressure variations generated underwater by the movement of objects. Recently, we have also conducted a research on high-precision, low cost piezoresistive MEMS-array pressure transmitters based on genetic wavelet neural networks for meteorological measurements [5]. It is noteworthy that the array of MEMS pressure sensors can reduce the intrinsic random error of the sensor due to creep by averaging measurements. However, according to the above literatures, we can see that the current sensor array is mainly used for sensing stress or pressure distribution, an exhaustive analysis considering the influences of doping concentration and the geometry of piezoresistors on optimizing the performance of the monolithically integrated array-type composite pressure sensor with repeating units for averaging measurements in terms of sensitivity, linearity as well as signal-to-noise ratio (SNR) has been rarely reported till now.

In this paper, in order to obtain high-performance array-type piezoresistive pressure sensors based on silicon on insulator (SOI) for radiosonde measurements of pressure, we investigate the design, optimization modeling, fabrication, measurement and temperature drift and nonlinear error compensation of the monolithically integrated MEMS pressure sensor array taking into account the balance between the low voltage noise and the high sensitivity as well as high linearity. Specifically, in contrast to pressure sensor arrays in existing literatures [5,9,18,19,20,21,22,23,24], this paper presents a novel array sensor structure integrated by two sets of four pressure sensors in different ranges onto the same chip to improve the overall performance. The array overcomes the weaknesses about the low sensitivity of large-range sensors to measure the small pressure and the high-pressure non-linear of low-range sensors to measure the high pressure. At the same time, accuracy of pressure sensor with each range can be improved through the average measurement. The high SNR is required for the faithful measurement of the small pressure differentials, and the different noise components commonly present with the piezoresistive-type sensors are carefully considered and modeled to improve the SNR [25,26,27,28,29]. It is noteworthy that the leakage current through the dielectric isolation instead of P-N isolation can be reduced and the operation temperature of the pressure sensor can be increased by using SOI material. Meanwhile, the fabrication process for the SOI-based array-type piezoresistive pressure sensor is also compatible with CMOS-MEMS technology [29]. However, the performance of array-type MEMS piezoresistive pressure sensor is highly dependent on temperature variation primarily due to the change in the piezoresistive coefficient with temperature. Temperature affects the piezoresistive coefficient through a change in the mobility and carrier concentration in the respective bands [25,30,31]. For the sake of eliminating the effects of temperature drift and nonlinear error as well as improving measurement accuracy, the wavelet neural network (WNN) improved by the genetic algorithm (GA) [5,32,33] is applied to achieve the temperature drift compensation in the study. Based on the structural characteristics of the array-type pressure sensor, the functional circuit and a graphical user interface (GUI) of data acquisition, digital communication and data storage are designed to realize the intelligent pressure sensor system.

## 2. Design Optimization of the Array-Type Piezoresistive Pressure Sensor and Basic Theory

### 2.1. Configuration of the Array-Type MEMS Pressure Sensor

**Figure 1:**
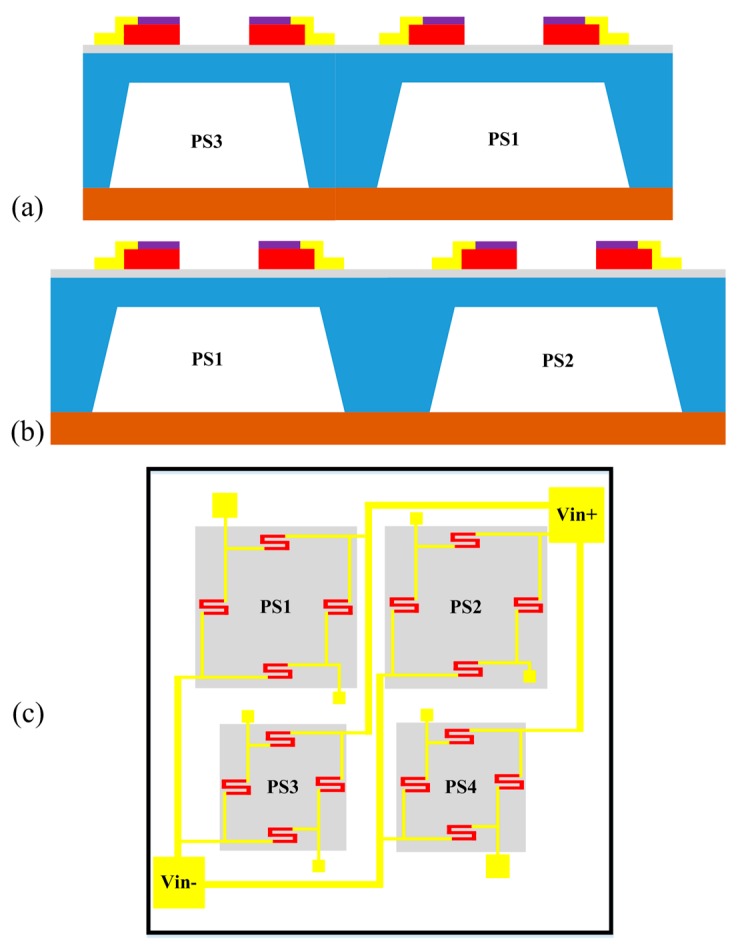
Schematic diagram of the array-type microelectromechanical system (MEMS) composite pressure sensor (**a**) side view of the array-type pressure sensor; (**b**) front view of the array-type pressure sensor; and (**c**) top view of the array-type pressure sensor.

Figure 1 displays the schematic of the proposed monolithically integrated array-type MEMS piezoresistive pressure sensor integrating four single pressure sensors. As can be seen in Figure 1a–c, they are divided into two ranges, each of which consists of two identical pressure sensors to reduce the random error by averaging measurements [5]. According to the statistical averaging theory, if n pressure sensors are used to measure the pressure at the same time, the overall average error becomes $1/\sqrt{n}$ of the original measurement error. Therefore, when one applies several sensors to constitute an array measurement to give the average output voltage of MEMS-array pressure sensors, it not only can reduce human errors and factory errors but also can weaken the intrinsic random error of the sensor due to creep.

In view of the compatibility of the manufacturing process, four single pressure sensors are integrated into the same pressure sensing chip by using the same mask with a variety of different size structures. The range of the PS1 and PS2 with the same structure is 0–50 kPa. Their diaphragm width is wide, and thus has a good sensitivity. The PS3 and PS4 have the same structure, and the range is 0–100 kPa. Due to the narrow diaphragm width, they have the good linearity. The array can overcome the weaknesses about the low sensitivity of large-range sensors to measure the small pressure and the high-pressure non-linear of low-range sensors to measure the high pressure. Thus, it is possible to achieve high linearity and sensitivity within the hybrid composite range (0–50 kPa and 50–100 kPa).

#### 2.1.1. Diaphragm Design

The pressure sensing diaphragm is utilized as a sensitive element of the MEMS pressure sensor, and its deformation and stress distribution affect the sensitivity and linearity. At present, most pressure-sensing diaphragms are rectangular, round, and square, of which the square diaphragm has the best sensitivity and its production process is relatively simple. For this reason, the array-type MEMS sensor’s diaphragm is designed into a square. The thickness of the sensing diaphragm will directly affect the performance. The thick diaphragm will make the sensor sensitivity decrease, while the thin one will lead to non-linear and bad anti-overload ability [12]. In the paper we choose 20 μm as silicon diaphragm thickness. To obtain good linear output, the deformation of the sensing diaphragm should meet the principle of small-deflection deformation at full-scale input [34].

On the one hand, the maximum deflection $\omega_{\max}$ of the elastic diaphragm is generally less than 1/5 of the diaphragm thickness *h* under external pressure *P*, which can be described as:

(1) $$\omega_{\max}=0.0151\frac{a^{4}P(1-\upsilon^{2})}{Eh^{3}}\leq \frac{1}{5}h$$

where Young’s modulus of silicon *E* = 170 GPa and the Poisson’s ratio υ = 0.278. According to Equation (1), we can obtain that a_1_ (the diaphragm length or width of PS1/PS2) and a_2_ (the diaphragm length or width of PS3/PS4) is less than 1680 μm and 1370 μm, respectively.

On the other hand, the stress and strain of the sensing diaphragm should maintain good linearity within the elastic deformation limit. For the square diaphragm, the ratio of length to thickness should obey the following relation:

(2) $$\frac{a}{h}\leq \sqrt{\frac{4}{3}\frac{\sigma }{P}}$$

where the failure stress value of silicon *σ* = 8 × 10^7^ Pa, and then the diaphragm length of PS1/PS2 and PS3/PS4 in the array-type sensor can be calculated as follows: *a*_1_ ≤ 900 μm and *a*_2_ ≤ 650 μm.

According to comparison of the above calculation results, *a*_1_ ≤ 900 μm and *a*_2_ ≤ 650 μm are chosen to ensure that the array-type silicon piezoresistive sensor has a good linearity.

##### 2.1.2. Design of Mask Window of the Silicon Cup

**Figure 2:**
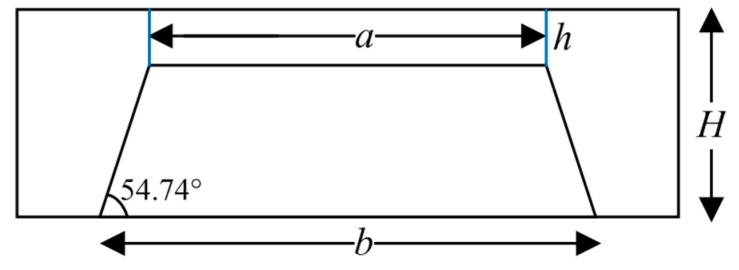
Schematic diagram of silicon cup structure of single MEMS pressure sensor.

The device layer thickness of a 6-inch (100) SOI wafer chosen in this paper is 1.5 μm, and the thickness of buried silicon oxide and the substrate silicon layer is 2 μm and 650 ± 15 μm, respectively. To obtain the elastic diaphragm with thickness of 20 μm, as shown in Figure 2, the silicon cup is corroded by the wet etching process. *H* denotes the total thickness of the SOI wafer, *h* is the thickness of the elastic diaphragm, *a* is the diaphragm length, *b* is the size of silicon cup window, *θ* is the angle between the (100) plane and the (111) plane. The calculation formula of silicon cup size is as follow:

(3) $$H-h=\frac{1}{2}(b-a)\tan\theta$$

According to Equation (3), we can know that *b*_1_ (the mask window length of PS1/PS2) = 1792 μm, and *b*_2_ (the mask window length of PS3/PS4) = 1542 μm. Thus, we design the size of single pressure sensor in the array is 3000 μm × 3000 μm.

##### 2.1.3. Design of the Piezoresistor

The *p*-type silicon piezoresistor is more conducive to reduce the effect of temperature on the sensor output signal than the *n*-type silicon, and it can improve the overall performance of the sensor. In this paper, the pressure-sensitive region is formed by implanting boron ions into the SOI wafer device layer, where the doping concentration should be as uniform as possible to ensure that the four resistance values of the Wheatstone bridge are the same and their sensitivity coefficients under stress are the same too. Then, the convex piezoresistor is formed by the photolithography etching process. In view of the significant effect of temperature on the performance of piezoresistive pressure sensor, we choose 1 mA bridge-arm current to supply the energy for the array-type sensor and reduce the temperature drift error. Generally, the maximum power consumption per unit area of piezoresistor is *p*_max_ =5 × 10^−3^ mW/μm^2^ [34], and the power consumption per unit area *p* is given by:

(4) $$p=\frac{I^{2}R}{lw}=\frac{I^{2}R_{S}}{w^{2}}$$

where *I* is the bias current applied to the piezoresistor, *R* is the resistance of the piezoresistor, *l* and *w* are the length and width of the piezoresistor, respectively, and *R_s_* is the square resistance. Therefore, the operating current passing through unit width of resistance strip can be expressed as:

(5) $$I_{\max}=\frac{I}{w}=\sqrt{\frac{p}{R_{S}}}$$

where the square resistance *R_s_* = *ρ*/*t*, the resistivity *ρ* = 0.04 Ω·cm, the piezoresistor thickness (after thermal oxidation process) *t* = 1 μm, thus the square resistance *R_s_* = 400 Ω/□. Based on Equation (5), we can obtain that the maximum operating current *I*_max_ = 0.112 mA/μm. Because the bridge arm current is 1 mA, so the width of the piezoresistor is *w* ≥ 8.9 μm. Due to the impact of the lithography technology, the narrower the piezoresistor, the greater the resistance error. Considering this situation, we make a wide piezoresistor as far as possible. In fact, the chip is also easy to cool down when the current passes through the wide piezoresistor. Therefore, combining with the actual process technology conditions of Jiangsu IntelliSense Technology Co., Limited (Jiangsu, China), we take 10 μm as the width of the piezoresistor. According to diaphragm area of sensor with the 0–100 kPa range which is relatively small and the stress distribution at the piezoresistor position, the length of the piezoresistor l is set to 150 μm, and the resistance is 6 kΩ. The sensitivity of the sensor is positively correlated with the stress value of the piezoresistor. The higher the stress value, the higher the sensitivity. A serpentine piezoresistor is used to make the piezoresistor more effective in the stress concentration area, as shown in Figure 1.

#### 2.2. Finite Element Analysis of the Array-Type MEMS Piezoresistive Pressure Sensor

##### 2.2.1. Structure Modeling and Simulation

**Figure 3:**
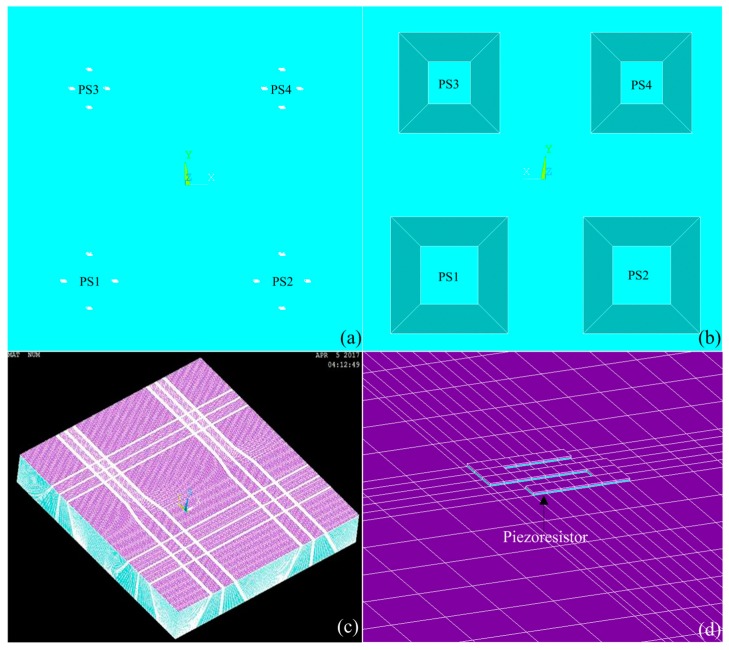
(**a**) Top view of finite element (FE) model of the array-type pressure sensor; (**b**) bottom view of FE model of the array-type pressure sensor; (**c**) three-dimensional FE mesh of the array-type pressure sensor; and (**d**) three-dimensional FE mesh of the piezoresistor area.

In order to make the proposed monolithically integrated array-type sensor have good linearity and sensitivity, we not only need to carry out the above-mentioned theoretical calculation and analysis of the discrete sensor, but also need to use the computer-aided software ANSYS (version 14.5, ANSYS Inc., Canonsburg, PA, USA) to establish a complex model of array-type sensor system and to implement finite element (FE) stress analysis, so that the array sensor parameters that meet the design requirements can be finally determined. The FE model of the array-type MEMS pressure sensor is plotted in Figure 3a,b, which is composed of the protective layer, silicon piezoresistors, the insulating layer and the silicon cup. Figure 3c,d presents three-dimensional finite element mesh of the array-type MEMS pressure sensor and the piezoresistor. In view of the small size of the piezoresistor bar compared to the whole model, the solid model of the array pressure sensor is cut by the working plane, and then the mesh partitioning is done by using high-precision hexahedral mesh generation method. To balance the calculation scale and accuracy, the piezoresistor bar and its nearby area is divided by a fine mesh.

**Figure 4:**
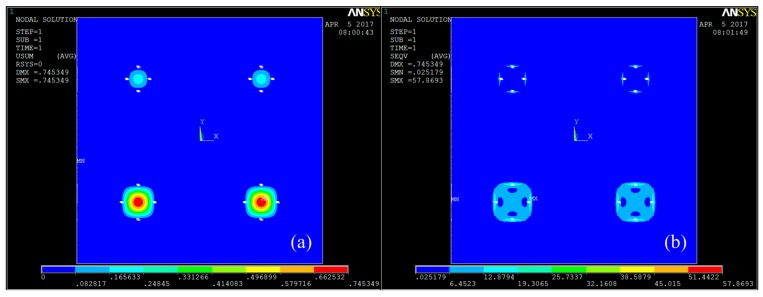
(**a**) Displacement cloud of array-type pressure sensor under 100 kPa; and (**b**) average stress distribution of array-type pressure sensor under 100 kPa.

Based on the above model, the stress distribution of the array can be solved by using ANSYS FEM. In the FE simulation process, the edge of array-type MEMS piezoresistive sensor is fixed with the silicon substrate, that is, the degree of freedom of the array-type sensor in the three directions of *x*, *y*, and *z* is limited, and then the external pressure is applied to the array top layer. Figure 4 shows the displacement cloud and the von Mises equivalent stress (SEQV) cloud of the array-type sensor under 100 kPa applied pressure. From the distributions of displacement and average stress of sensing diaphragm, it can be seen that the largest deformation occurs in the center of the diaphragm while the largest stress is in the middle of the edge of diaphragm (the position of the piezoresistor). This is exactly what we expected. When the material characteristics (such as doping concentration) of the piezoresistor are determined, the sensitivity of the array sensor is only related to the stress value of the piezoresistor bar. The greater the stress value the array gets, the higher the sensitivity the sensor has. In addition, it is found that in the case of the same thick diaphragm, the stress value of piezoresistor of the PS1/PS2 pressure sensor is obviously larger than that of PS3/PS4, which can result in a more significant sensitivity. In the following sections, we present an analysis of the array-type sensor pressure sensitivity, voltage noise and the signal-to-noise ratio.

##### 2.2.2. Theoretical Analysis of Sensitivity and SNR of Array-Type Pressure Sensors

The sensitivity is a critical indicator of the piezoresistive pressure sensor. It relies on the initial zero-stress resistance *R* of the piezoresistors, which is determined by its size and doping concentration as well as temperature [26,29,30]. For a piezoresistor with area *A_R_*, the piezoresistive pressure sensitivity *S* is given by:

(6) $$S=\frac{\Delta R/R}{\Delta P}=\frac{1}{A_{R}\Delta P}\int_{0}^{A_{R}}{(\pi }_{L}\sigma_{L}+\pi_{T}\sigma_{T})\partial A$$

where Δ*R* represents the resistance change of the piezoresistor under the differential stress Δ*P*. π*_L_* and π*_T_* refer to the longitudinal piezoresistive coefficient (the current direction is consistent with the stress direction in the piezoresistor) and transverse piezoresistive coefficient (the current direction is perpendicular to the stress direction), respectively, while *σ_L_* and *σ_T_* are the longitudinal and transverse stress in the piezoresistors formed by the external pressure Δ*P*.

According to Equation (6), for investigating the relationship between the sensitivity and doping concentration as well as temperature, the dependence of the piezoresistive coefficient (π) as functions of the doping concentration (*n*) and temperature (*T*) is considered. The piezoresistive coefficient π(*n*,*T*) of the silicon piezoresistor obeys to the following relation [26,29]:

(7) $$\pi (n,T)=\pi (n_{0},T_{0})\frac{300}{T}\frac{F_{-1/2}(E_{F}/K_{B}T)}{F_{1/2}(E_{F}/K_{B}T)}$$

The coefficient π(*n*_0_,*T*_0_) denotes the piezocoefficient value for the silicon of low doping concentration (*n*_0_) at room temperature (*T*_0_). The Fermi integral is the function of temperature (*T*) and the Fermi energy (*E_F_*) which is determined from *n*, and *K_B_* stands for the Boltzmann constant. It is found that the piezoresistive effect significantly decreases at high temperature and doping concentration which is mainly associated with carrier-phonon scattering and carrier-carrier scattering [30,35].

**Figure 5:**
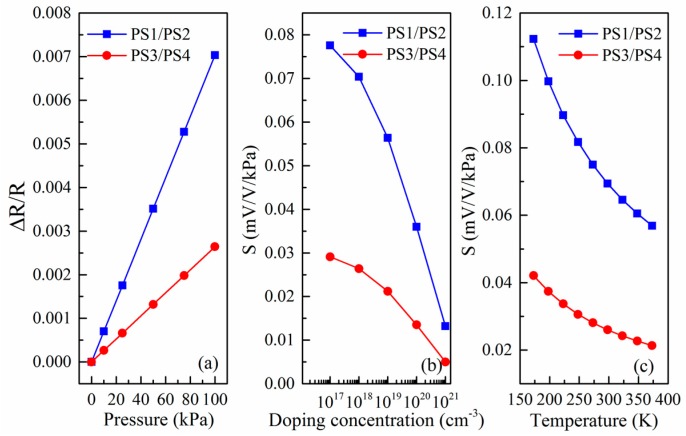
(**a**) Relative resistance change as a function of the applied pressure for array-type sensor; (**b**) variation of the sensitivity of array-type pressure sensor with the doping concentration; and (**c**) variation of the sensitivity of array-type pressure sensor with the temperature.

In terms of finite element stress analysis and Equation (6), the sensitivities of PS1/PS2 and PS3/PS4 are calculated and analyzed by extracting the transverse and longitudinal stress of the piezoresistor bar at room temperature, respectively. Figure 5a demonstrates the change in resistance with the applied pressure in the range of 0 to 100 kPa. It is observed that PS1/PS2 sensor has sensitivity of 0.065 mV/V/kPa which is about 2.5 times more than one of the PS3/PS4 pressure sensor. It is worth noting that the increase in sensitivity is mainly due to the increase in diaphragm size of PS1/PS2 can lead to more stress. Based on microscopic piezoresistive model we previously proposed [30], the relationship between sensitivity and doping concentration of array-type piezoresistive sensor is also calculated by using Equations (6) and (7). As can be from Figure 5b, the sensitivity decreases monotonically with the doping concentration, which is mainly owing to the piezoresistive coefficient decreases with increasing doping concentration. Therefore, for the design of high-sensitivity miniature pressure sensor, the doping concentration should not be too high. Meanwhile, the doping concentration cannot be too low. On the one hand, it is difficult to form an Ohmic contact when the doping concentration is low. On the other hand, the sensor noise and temperature drift increase with the doping concentration decreasing, thus affecting the overall SNR [26,29]. So, the doping concentration value has a preferred value, and the selection of the doping concentration should be comprehensively considered. Furthermore, Figure 5c gives the theoretical calculation results for the relationship between the sensitivity and the temperature. It is similar for PS1/PS2 and PS3/PS4, and the sensitivity monotonically decreases with the temperature increases, which is mainly attributed to the piezoresistive coefficient of the silicon piezoresistor decreases with increasing the temperature [30]. A clear nonlinear relationship is observed as the temperature increases, and the temperature coefficient of sensitivity (TCS) extracted by linear fitting is around −2.499 × 10^−4^ K^−1^ and −9.502 × 10^−5^ K^−1^ for PS1/PS2 and PS3/PS4, respectively.

As we all know, there is noise in the output signal of the sensor. At present, the sensitivity of the sensor is greatly improved, but the noise may be amplified also. In the case, the SNR has become an important factor reflecting the performance of pressure system. Combined with the relevant noise model [26,29,36,37,38], detailed analysis of the array-type pressure sensor noise and SNR are given. For the MEMS piezoresistive pressure sensor, the noise power spectral density $V_{\mathrm{noise}}^{2}$ of the piezoresistor is mainly composed of Johnson noise power spectral density $V_{J}^{2}$, flicker (1/*f*) noise power spectral density $V_{f}^{2}$ and Brownian noise power spectral density $V_{B}^{2}$ [25,26,29,36,37,38], which can be expressed as:

(8) $$V_{\mathrm{noise}}^{2}=V_{J}^{2}+V_{f}^{2}+V_{B}^{2}$$

where Johnson noise generates in resistors owing to random motion (thermal agitation) of carriers and is independent of frequency. The dominant 1/*f* noise source in silicon piezoresistors is Hooge noise [8,36], which is a fluctuation in resistor conductance caused by drawbacks in the bulk of the material. Different from Johnson noise, it is a conductivity voltage noise that depends on the bias voltage. The Brownian noise of the system is introduced by mechanical fluctuations of the pressure-sensitive membrane from a Brownian force and is a direct physical analog of Johnson noise related to electrical resistance. In low frequency environment, Brownian noise can be neglected compared to the Johnson noise and flicker noise [25]. The Johnson noise and flicker noise power spectral density can be expressed as:

(9) $$V_{J}^{2}=4K_{B}TR(f_{\max}-f_{\min})$$

(10) $$V_{f}^{2}=\frac{\alpha }{N}{\left(IR\right)}^{2}\ln(\frac{f_{\max}}{f_{\min}})$$

where *R* is the resistance of the piezoresistor with the total free carrier number *N*, *f* is the frequency (*f*_min_ = 10 Hz, *f*_max_ = 1 kHz), *α* is the Hooge factor, which is the key parameter that characterizes 1/*f* noise and is between 3.2 × 10^−6^ and 5.7 × 10^−6^ in single crystal silicon.

Signal-to-noise ratio (SNR) is a key parameter that reflects the performance of pressure sensor. For the case when the constant current is applied, the final expression of SNR for a given geometry and doping in a bandwidth from *f*_min_ to *f*_max_ is as follow:

(11) $$SNR=20\log\frac{I\pi \sigma R}{\sqrt{4K_{B}TR(f_{\max}-f_{\min})+\alpha I^{2}R^{2}/(n_{p}lwt)\ln(f_{\max}/f_{\min})}}$$

where *w* and *t* are the width and thickness of the piezoresistor, and *n_p_* is the carrier concentration.

**Figure 6:**
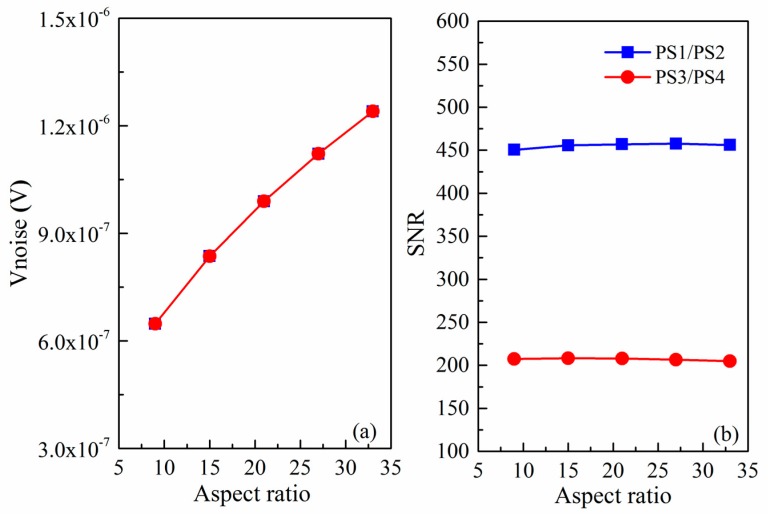
(**a**) Variation of the voltage noise with the aspect ratio; and (**b**) variation of SNR with the aspect ratio.

In the following section, the influencing factors of noise and SNR of array-type pressure sensor are analyzed by using the above noise models (Equations (8)–(11)). The variation of the voltage noise and SNR with the aspect ratio of the piezoresistor is plotted in Figure 6. For the 1 mA constant-current and 1 × 10^18^ cm^−3^ doping concentration case, Figure 6a reveals that the noise voltage of the sensor is proportional to the aspect ratio of the piezoresistor and the noise curve of PS1/PS2 coincides with that of PS3/PS4. However, SNR does not follow a similar law. As can be seen in Figure 6b, the SNR is relatively stable under different aspect ratio, so we can ignore the effect of the aspect ratio when we design the sensor. It is also observed that the SNR of PS1/PS2 in the array is more than 2 times higher than that of PS3/PS4.

**Figure 7:**
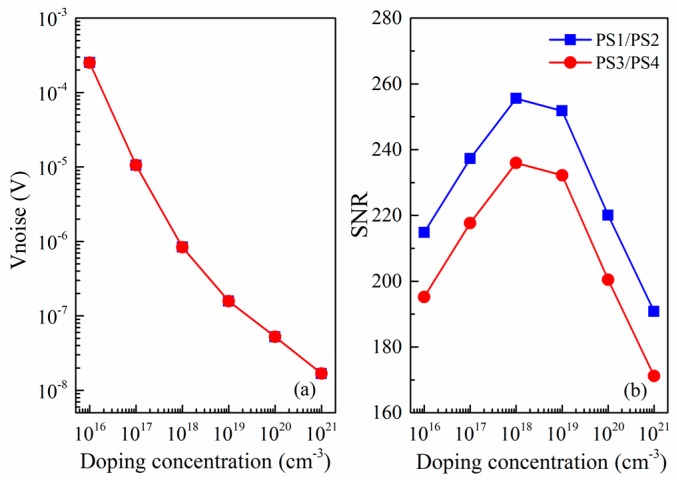
(**a**) Noise as function of doping concentration; and (**b**) SNR as function of doping concentration.

Figure 7 displays the relationship between the doping concentration and the noise as well as SNR. It can be found from Figure 7a that the noise voltage is inversely proportional to the doping concentration, and the higher the doping concentration is, the smaller the noise is. In addition, as can be seen in Figure 7b, the SNR firstly increases with doping concentration increasing because the flicker noise voltage varies inversely. However, the SNR subsequently decreases since the Johnson noise voltage tends to dominate and piezoresistive coefficient is also reduced. It is apparently noted that the maximum SNR is located around 1 × 10^18^ cm^−3^ doping concentration. Considering the good Ohmic contact and the low power consumption of array-type pressure sensor, *p*-type implantation of 1 × 10^18^ cm^−3^ is then chosen. Besides, we can also find that the SNR of PS1/PS2 pressure sensor has been improved compared to PS3/PS4 pressure sensor. In short, our work shows that we can optimize the design to reduce the noise and improve the signal-to-noise ratio of piezoresistive pressure sensor. We hope that the results of these theories can be helpful for future design of experiment methods to achieve the most optimized array-type pressure sensor.

## 3. Fabrication Process of Array-Type MEMS Piezoresistive Sensor

Based on the structural design and simulation analysis, the layout and process flow of the pressure chips are designed and then the array-type pressure sensor are fabricated and packaged in Jiangsu IntelliSense Technology Co., Limited (Jiangsu, China).

### 3.1. Process Flow

**Figure 8:**
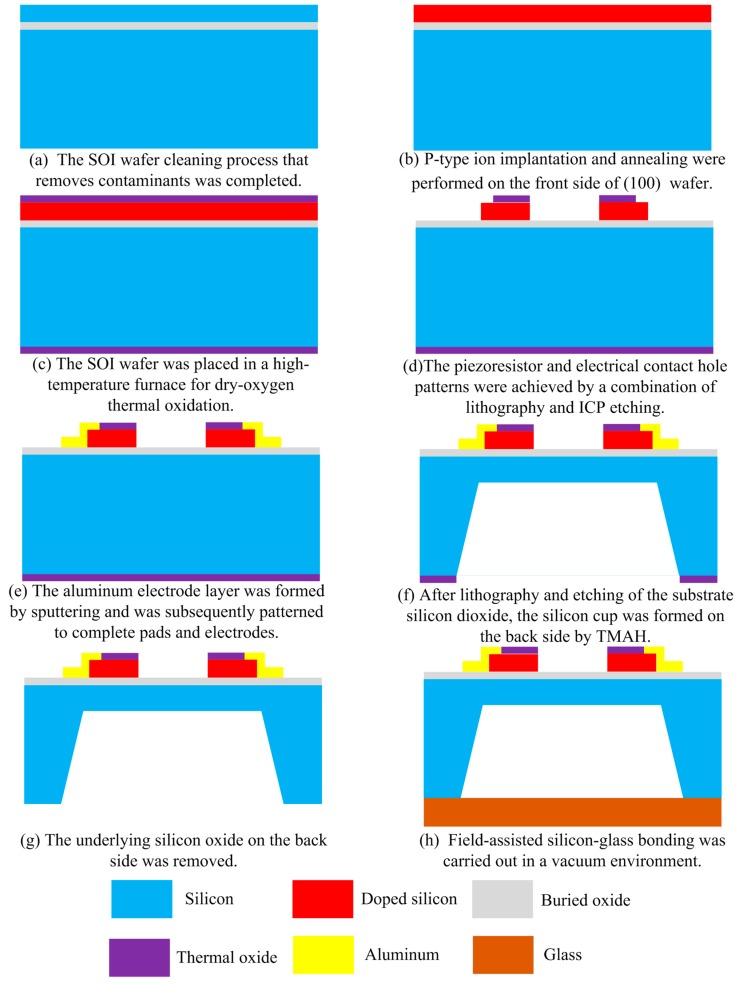
Process flow of the proposed array-type piezoresistive pressure sensor (the drawing is not to scale). (**a**) the silicon on insulator (SOI) wafer cleaning process that removes contaminants was completed; (**b**) *p*-type ion implantation and annealing were performed on the front side of (100) SOI wafer; (**c**) The SOI wafer was placed in a high-temperature furnace for dry-oxygen thermal oxidation; (**d**) the piezoresistor and electrical contact hole patterns were achieved by a combination of lithography and ICP etching; (**e**) the aluminum electrode layer was formed by sputtering and was subsequently patterned to complete pads and electrodes; (**f**) after lithography and etching of the substrate silicon dioxide, the silicon cup was formed on the back side by tetramethylammonium hydroxide (TMAH); (**g**) the underlying silicon oxide on the back side was removed; (**h**) field-assisted silicon-glass bonding was carried out in a vacuum environment.

The proposed array-type MEMS piezoresistive pressure sensor with optimized size and doping concentration was fabricated with *p*-type (100) SOI wafer by using the standard CMOS lithography process and anisotropic wet-etch release process. The double-sided polished SOI wafer has a 2 μm buried oxide (BOX) layer and a 1.5 μm top silicon layer. Figure 8a–h briefly shows the fabrication process of this monolithically integrated array-type pressure sensor. The specific process mainly consists of the following steps:

Step1: wafer preprocessing. The SOI wafer was placed in 1NH_4_OH: 1H_2_O_2_: 5H_2_O solutions at 80 °C for removal of the particles and in 1HCl: 1H_2_O_2_: 6H_2_O solutions at 80 °C for removal of inorganic contamination, and then repeatedly cleaned with deionized water. Finally, the cleaned wafer was placed in diluted HF solution to remove the natural oxidation of its surface;

Step2: ion implantation and annealing. The 1 × 10^18^ Dose/cm^3^ Boron ions were injected to SOI device layer by 20 keV energy, then SOI wafer was placed in a 1000 °C annealing furnace for 30 min to distribute the boron particles evenly to ensure that the number of carriers is same as the injected dose;

Step3: thermal oxidation. The SOI wafer was placed in a high-temperature furnace at 1000 °C for dry-oxygen oxidation, and the oxidation thickness is about 1 μm, which can be used as a passivation layer for the device layer;

Step 4: lithography and etching. The piezoresistor and electrical contact hole patterns were achieved one after the other by a combination of lithography and etching. Spin-on deposition and baking were firstly performed on an EVG^®^ 150 automated spin-/spray-coating equipment. Subsequently, the wafer is exposed for 50 s and immersed in Microposit^®^ MF-312 developer (1:1 dilution in deionized water, Shipley Contracting Corp., Burlington, IA, USA.) for 90 s at room temperature. After baking, we succeeded in the vertical and smooth dry etching of thermally grown SiO_2_ and silicon at low ion energy by introducing an inductively coupled plasma (ICP) etching process. Afterwards, the wafer was cleaned with acetone at 65 °C to completely remove the photoresist and then rinsed 2–3 times with deionized water;

Step 5: sputtering aluminum. The aluminum electrode layer was formed with a thickness of 1.5 μm by radiofrequency (RF) magnetron sputtering in an ATC Orion-8 UHV system (AJA International, Inc., Scituate, MA, USA) at room temperature, and was subsequently patterned to complete pads and electrodes. At the same time, ohmic contact between aluminum and piezoresistive was achieved;

Step 6: silicon cup formation. After lithography and etching of the substrate silicon dioxide, the silicon cup window was formed on the back side, and then tetramethylammonium hydroxide (TMAH) by addition of parts of 3% ammonium persulfate with temperature of 80 °C was used as the etching solution for the silicon cup. After etching for 12 h, the silicon cavity was rinsed with dilute hydrochloric acid;

Step 7: oxygen removing. The silicon dioxide etching solution was prepared using hydrofluoric acid (50%) and ammonium fluoride (40%) with a weight ratio of 1:7 to remove the underlying silicon oxide on the back side;

Step 8: anodic bonding. The SOI wafer was placed on a heat-resistant glass (Pyrex 7740) under vacuum, and the temperature was heated to 400 °C, and then a forward voltage of 1000 V was applied to the wafer. After a few minutes of bonding, the current was dropped to zero and the electrostatic bonding was completed. Finally, separate monolithically integrated array-type sensor chips were cut from the wafer by using a dicing saw.

**Figure 9:**
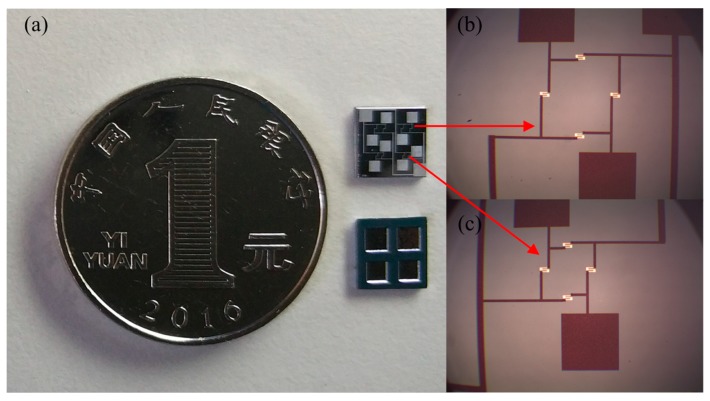
(**a**) Physical map of array-type pressure sensor; (**b**) a photograph of the fabricated PS1/PS2 pressure sensor; and (**c**) a photograph of the fabricated PS3/PS4 pressure sensor.

The physical map of the monolithically integrated array-type composite pressure sensor chip is shown in Figure 9a, and the optical microscope photographs of PS1/PS2 and PS3/PS4 sensors are shown in Figure 9b,c, respectively.

### 3.2. Sensor Chip Package

**Figure 10:**
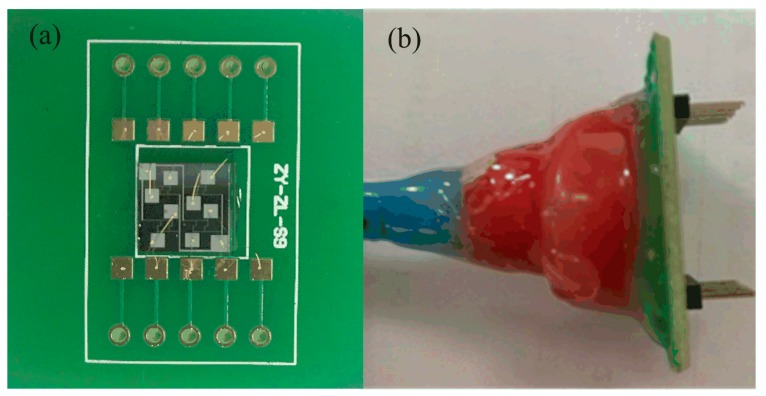
(**a**) Photograph of the pressure-welded array-type piezoresistive pressure sensor on the printed circuit board (PCB); (**b**) photograph of the packaged array-type piezoresistive pressure sensor.

After the monolithically integrated array-type pressure sensor chip was manufactured by the above process, it was pressure-welded and packaged on the printed circuit board (PCB) for calibration and performance evaluation. As shown in Figure 10a, the array-type sensor chip was firstly attached to the PCB by die-bonding process, and then gold wires were used to connect the chip pins with the electrical pads on the PCB. Finally, the chip is sealed with a circular plastic box, as illustrated in Figure 10b, the sealing effect is good after many tests have been taken.

## 4. Hardware Design and Software Design

### 4.1. Hardware Design of Array-Type Intelligent Pressure Sensor System

**Figure 11:**
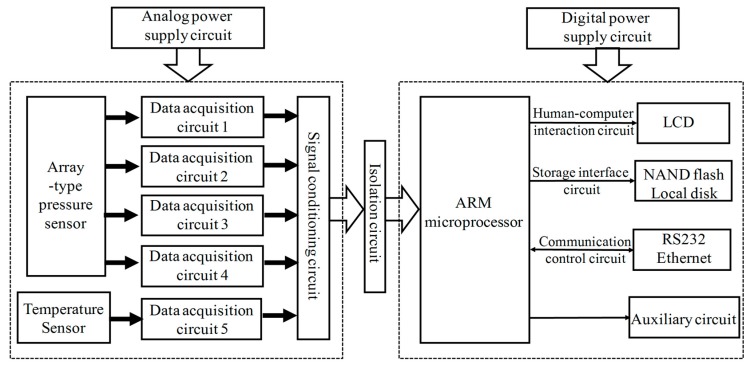
The systematic block diagram for hardware implementation of the array-type MEMS intelligent pressure sensor system.

**Figure 12:**
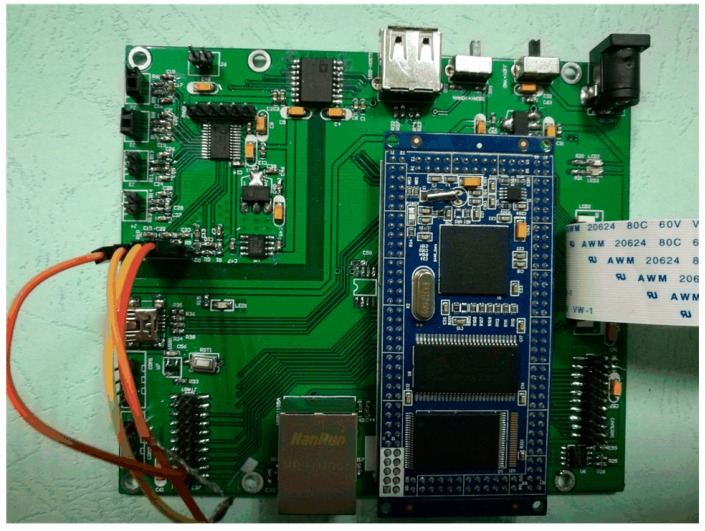
Photograph of the designed hardware circuit system of the array-type MEMS intelligent pressure sensor system.

Figure 11 provides the systematic diagram for hardware implementation of the complete scheme. Hardware circuits of the array-type intelligent pressure sensor are mainly composed of analog and digital circuits. Analog circuits consist of power supply circuit, isolated communication circuit, data acquisition circuit and signal conditioning circuit. They mainly implement the acquisition and condition of pressure and temperature sensor signals for compensation. Digital circuits consist of a power supply circuit, Samsung S3C2440 ARM microprocessor, human-computer interaction circuit, storage circuit and network communication circuit. They mainly achieve digital signal processing, including compensation algorithm, data communication and storage. The photograph of the designed hardware circuit system is presented in Figure 12.

### 4.2. Software Design of Array-Type Intelligent Pressure Sensor System

**Figure 13:**
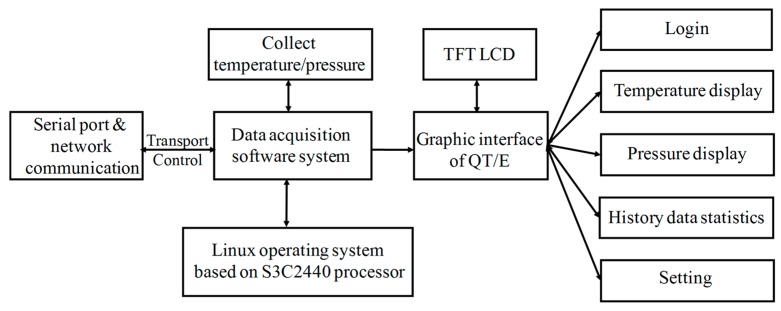
Software system framework of the array-type intelligent pressure sensor.

**Figure 14:**
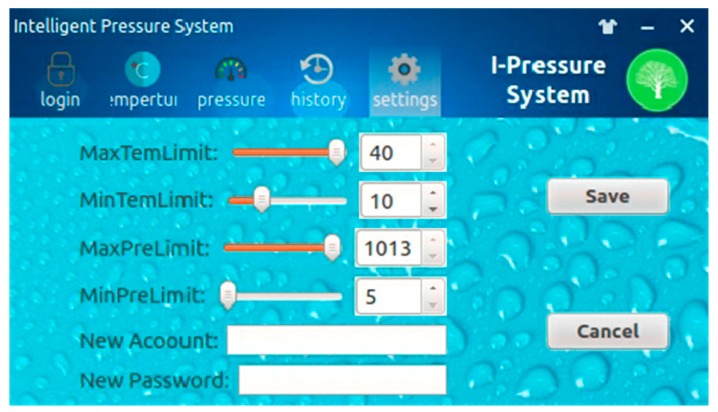
Graphical user interface for human-computer interaction.

The software system of the array-type intelligent pressure sensor was constructed with QT/E graphical library under Linux operating system, which was then transplanted on Samsung S3C2440 advanced RISC machines (ARM) chip. The software system framework is plotted in Figure 13. The entire software system includes three major entities, as follows: data acquisition, data transmission and control, and QT/E applications. Data acquisition is mainly done by writing AD7794 (Analog Devices, Inc., Norwood, MA, USA) device drivers under Linux operating system to collect the pressure and temperature data. Data transmission and control system transmits the collected data to the client through the serial port and network communication mode and accepts the control commands of the client. The QT/E applications mainly complete the human-computer interaction graphic display, data management and system setting. The graphic display refers to the real-time display of the collected data on the thin-film transistor (TFT) liquid-crystal display (LCD) in the form of numerical values and curves. The data management refers to achieving query, storage, deletion, and analysis of the collected data. The system refers to setting the pressure and temperature thresholds, user name and password. Graphical user interface for human-computer interaction of the array-type intelligent pressure sensor system is given in Figure 14.

## 5. Experimental Section and Discussion

In this section, the temperature drift characteristic of the array-type pressure sensor is evaluated. The improved wavelet neural network based on genetic algorithm (GA-WNN) is then used to fuse the data to compensate for the measurement error of the sensor. It is further verified that the idea of the array-type sensor which has both high sensitivity and linearity is reasonable. Finally, the performance index of array-type MEMS piezoresistive pressure sensor system is given.

### 5.1. Experimental Setup

**Figure 15:**
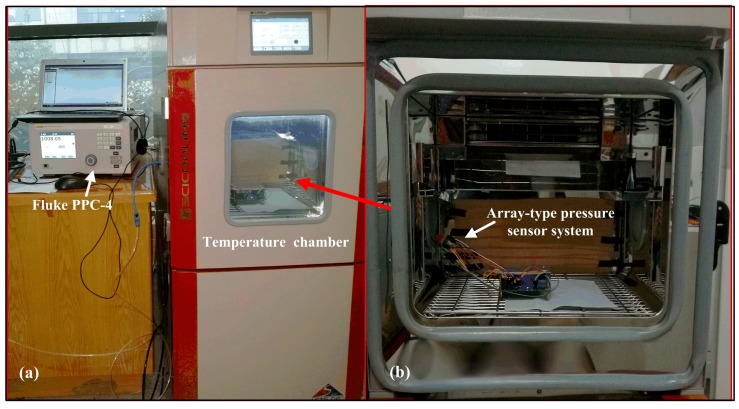
Photographs of calibration and test platform for the array-type pressure sensor system.

Experimental calibration and test system of the array-type pressure sensor is divided into the following modules: (1) Pressure control module. A Fluke PPC-4 standard pressure generator (Fluke Corporation, Everett Reed, WA, USA) with 4 ppm stability and ±0.008% accuracy is utilized to control the high-pressure gas cylinder to provide the sensor with precise pressure values from 0 kPa to 100 kPa; (2) Temperature control module. The constant temperature chamber manufactured by China-Scicooling Science and Technology Limited Company (Beijing, China) is applied to control the ambient temperature at where the pressure sensor is located. The calibration and test experimental setups are displayed in Figure 15. The calibration procedure is as follows [5,29]: Firstly, the array-type MEMS pressure sensor system without calibration is put into the constant temperature chamber. The airway of Fluke PPC-4 pressure generator is connected to the air inlets of the array-type sensor. At the same time, the pressure generator is used to produce the standard calibration pressure that we want to get, such as 0 kPa, 10 kPa, 20 kPa, 30 kPa and so on. In addition, then the average pressure voltage value of PS1/PS2 and the average pressure voltage value of PS3/PS4 and temperature voltage value are measured by using our array-type pressure sensor and temperature sensor, respectively. Secondly, the temperature in the experimental chamber is changed from −20 to 20 °C to facilitate calibration pressure and temperature drift compensation. Thirdly, the data fusion algorithm based on the GA-WNN is introduced to establish the function relationship between the standard calibration pressures obtained by the Fluke pressure generator and the pressure voltage values as well as the temperature values obtained by our sensor system in wide ranges of temperature and pressure. Fourthly, the GA-WNN algorithm and the function relationship which minimizes the sensor errors due to the temperature drift and the nonlinear effect is written into the embedded system that allows the array-type intelligent pressure sensor real-time and display pressure data online. Finally, we conduct the experiments to test the prediction performance of the GA-WNN algorithm.

### 5.2. Array-Type Sensor Output Result

The calibration output voltages of the MEMS array-type sensors (PS1/PS2 and PS3/PS4) under different pressure and temperature (the calibration sample data) for training GA-WNN compensation algorithm are experimentally measured, which are listed in Table 1 and Table 2, respectively. The output standard pressure of the Fluke pressure generator is marked as *P*, and the *U_p_* represents the average output voltage of the MEMS array-type sensors. From Table 1 and Table 2, it is found that the *U_p_* changes with temperature *T* and hence there is a drift in the array-type sensor output characteristics. The temperature drift can especially cause a few mV errors at full scale. Hence, the pressure sensor is influenced by the temperature. 

**Figure 16:**
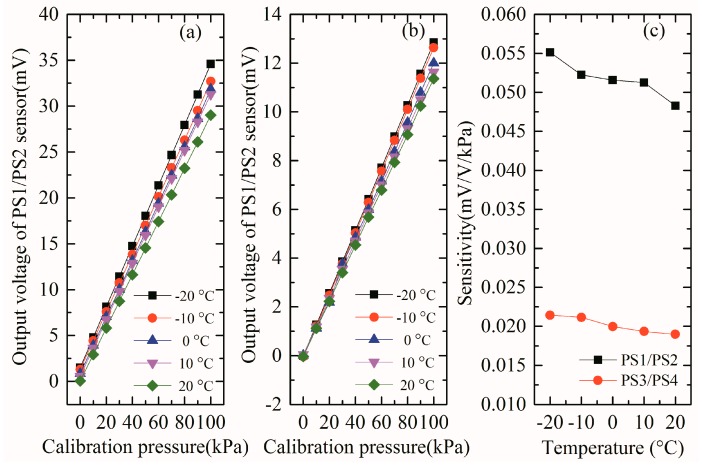
(**a**) The relationship between the output voltages of the PS1/PS2 sensor and the standard pressures; (**b**) the relationship between the output voltages of the PS3/PS4 sensor and the standard pressures, and (**c**) the variety of the sensitivity of array-type pressure sensor with temperature.

According to measurement data in Table 1 and Table 2, we firstly evaluate the characteristic of array-type MEMS sensor, and the nonlinear error of the PS1/PS2 and PS3/PS4 sensors is 0.286% and 0.193%, respectively. Based on Equation (6), the obtained average sensitivity of PS1/PS2 and PS3/PS4 sensors at different temperatures is 0.052 mV/V/kPa and 0.020 mV/V/kPa respectively, which is basically consistent with the theoretical analysis. The nonlinear error of PS1/PS2 is more than that of PS3/PS4 and the sensitivity of PS1/PS2 is much more than that of PS3/PS4 from 0 kPa to 100 kPa. The above results indicate the basic theory that the low-range pressure sensor has high sensitivity and poor linearity, while the large-range pressure sensor has bad sensitivity and good linearity. Then the temperature drift characteristics of the array pressure sensor are analyzed and studied. According to the relationship between the output voltages of the array sensor and the standard pressures at different temperature conditions in Table 1 and Table 2, we draw the curves, as presented in Figure 16a,b. It can be found that the voltage values at different temperatures are basically linear with the standard pressure values, and the output voltages of the array decreases significantly with increasing ambient temperature, which shows a well-known temperature drift phenomenon. In addition, it is not difficult to find from Figure 16c that the sensitivity of the array-type sensor decreases as the temperature increases between −20 °C and 20 °C. In particular, the sensitivity of PS1/PS2 reduces from 0.055 mV/V/kPa to 0.048 mV/V/kPa, while that of PS3/PS4 reduces from 0.022 mV/V/kPa to 0.019 mV/V/kPa. TCS extracted by linear fitting of PS1/PS2 is −1.467 × 10^−4^ K^−1^ and that of PS3/PS4 is −6.721 × 10^−5^ K^−1^, which is consistent with the theoretical calculation. It is noteworthy that TCS of PS1/PS2 is different from that of PS3/PS4 which is mainly because they have different stresses under the same pressure. According to Equations (6) and (7), we can see that the temperature mainly affects the piezoresistive coefficient, but the sensitivity is also a stress-related quantity, so even though the two sensors have the same tendency of the piezoresistive coefficient with temperature, different stresses can make the trend of sensitivity with temperature different.

On the other hand, to calculate and evaluate hysteresis and repeatability errors, we repeatedly measure the forward and reverse travel of array-type MEMS pressure sensor. Each pressure test point of the forward travel (*U*_inc_) rises from 0 kPa to itself, and each pressure test point of the reverse travel (*U*_dec_) falls from 100 kPa to itself [5]. As we all known, the hysteresis and repeatability are related to the creep of the material itself and residual stresses in the package [39], here we take PS3/PS4 sensor as an example. The experiment results without compensation at 20 °C are listed in Table 3. According to the definition, the repeatability and hysteresis errors can be calculated [5]. The repeatability error is 0.158% and the hysteresis error is 0.203%, which means that they are not low enough. They will affect measurement accuracy of the array-type sensor. To reduce these errors, we need to use GA-WNN compensation algorithm to correct them. Despite this, these errors can be fundamentally eliminated only by improving the level of the manufacturing process and material stability.

### 5.3. Data Fusion Using GA-WNN Compensation Algorithm

#### 5.3.1. GA-WNN Algorithm Overview

To reduce temperature drift, nonlinear and hysteresis errors, GA-WNN algorithm is used to data fusion of output of sensor in the paper. The basic idea of GA-WNN algorithm is as follows: the genetic algorithm is firstly utilized to construct a continuous evolutionary sequence, and the basic solution is obtained according to the evaluation method. Then the basic solution is applied as the initial state of neural network to carry out wavelet neural network training, which eliminates the random network initialization and makes the wavelet network easier and faster to get the optimal solution of the problem. The details of the GA-WNN have been described in the previous paper [5]. The key problem in GA-WNN algorithm is the selection of coding procedure and the determination of fitness function with the optimum parameters. In this paper, the weighting, the translation, and the scaling factors are taken as chromosomes in the learning process of the GA-WNN algorithm and the appropriate fitness function is selected. In addition, then the genetic algorithm is iterated until the optimal value is obtained in the network convergence. The specific implementation steps are as follows:

Step 1: The initial population is randomly generated by real coding. Each individual in the population represents a wavelet neural network structure and the connection parameters (the connection weights, the scaling factor, and the translation factor) corresponding to the structure, and the maximum number of iterations *M* is set.

Step 2: The input training samples are provided to calculate the error function of the wavelet neural network corresponding to each individual in the population, and the corresponding fitness value is obtained.

Step 3: Determining whether the fitness value of the new generation of population satisfies the optimization goal $F=(\sum_{K=1}^{M}{\left(Y_{K}-y_{K}\right)}^{2}{)}^{-1}>E_{\mathrm{goal}\text{\_}\mathrm{error}}^{-1}$, wherein the inverse of the sum of squares of errors between the desired output value *Y_K_* of the wavelet network and the actual output value *y_K_* is used as the fitness function, and $E_{\mathrm{goal}\text{\_}\mathrm{error}}$ is the target error of the network, if one can find a satisfactory individual then go to Step 6, otherwise go to Step 4.

Step 4: The fitness is sorted, and then it is copied to the next generation individuals in accordance with the best individual preservation strategy and the fitness proportional selection mechanism.

Step 5: The current generation population is subjected to crossover and mutation operations to get the next generation. To avoid falling into the local minimum solution, the crossover and the mutation rates will change with the fitness function value during the computation process. One checks whether the iteration reaches the maximum iteration *M*, if yes turn to Step 6, otherwise turn to Step 2.

Step 6: The parameters corresponding to the best individuals in the final population are decoded to obtain the optimal wavelet neural network structure and corresponding parameters.

In the following section, we discuss the compensation effect of the GA-WNN algorithm for array-type MEMS piezoresistive sensor system in detail.

#### 5.3.2. Analysis of Compensation Results Based on GA-WNN Algorithm

MATLAB (version R2013b, MathWorks, Natick, MA, USA) is applied to establish a data fusion model based on the GA–WNN algorithm and process these training sample data. The training data must be pre-processed to prevent nodes quickly reaching a saturated state and becoming unable to continue learning. According to the “mapminmax” function of MATLAB, the normalized formula is as follows:

(12) $$y=\frac{\left(y_{\max}-y_{\min})\times (x-x_{\min}\right)}{\left(x_{\max}-x_{\min}\right)}+y_{\min}$$

If the data are normalized to (−1, 1), *y*_max_ is 1 and *y*_min_ is −1. *x* is the output value of array-type pressure sensor at a reference temperature, while *x*_max_ and *x*_min_ are the maximum and the minimum output values, respectively. Based on this formula, the training data in Table 1 and Table 2 can be normalized. This paper sets the input layer with 2 nodes (corresponding to the temperature signal and pressure signal without compensation), the hidden layer with 11 nodes, and the output layer with 1 node (corresponding to the pressure output after compensation). Crossover probability is 0.75, mutation probability is 0.08, and the initial population size is 250. The termination condition of the genetic algorithm is that the fitness value is greater than 0.9. The momentum factor is 0.01, and the learning rate is 0.001. When the sum of square-error is 0.0001, the wavelet neural network ends. After these parameters are set up, the normalized input and output data as the sample can be put into the network for training.

**Figure 17:**
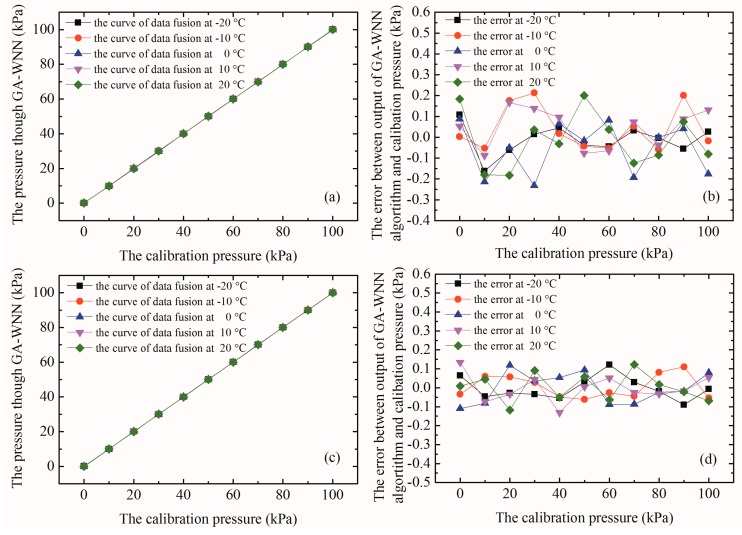
(**a**) The relationship curves between the corrected pressures of PS1/PS2 sensor and the calibration pressures with pressure from 0–100 kPa; (**b**) the absolute error between the corrected pressures of PS1/PS2 sensor and the calibration pressures at different temperature; (**c**) the relationship curves between the corrected pressures of PS3/PS4 sensor and the calibration pressures with pressure from 0–100 kPa; and (**d**) the absolute error between the corrected pressures of PS3/PS4 sensor and the calibration pressures at different temperature.

Figure 17 summarizes experimental results of MEMS array-type sensor after the temperature and nonlinear compensation through the GA-WNN data fusion algorithm. Specifically, Figure 17 exhibits calibrated pressure values of the array-type pressure sensor processed by the GA-WNN algorithm and the error between the calibrated output and the standard pressure. It is observed from Figure 17a,c that each calibration pressure point basically does not change with temperature in the range of 0–100 kPa, indicating that the temperature drift is well suppressed. Moreover, the curves coincide together and the linearity of array-type sensor after data fusion has become better. Figure 17b,d illustrate the absolute error between the output pressure that is compensated by the GA-WNN data fusion algorithm and standard calibration pressure obtained by Fluke pressure generator. Obviously, the error is significantly reduced. Then, we evaluated the linearity and temperature drift characteristics of the array-type pressure sensor. The nonlinear errors of the PS1/PS2 and PS3/PS4 decreased from 0.286% and 0.193% to 0.178% and 0.130%, respectively. The temperature coefficient of sensitivity of the PS1/PS2 and PS3/PS4 is reduced to −4.10 × 10^−6^ K^−1^ and −1.41 × 10^−6^ K^−1^, respectively. It is shown that GA-WNN algorithm has a good temperature compensation effect for the array-type sensor.

**Figure 18:**
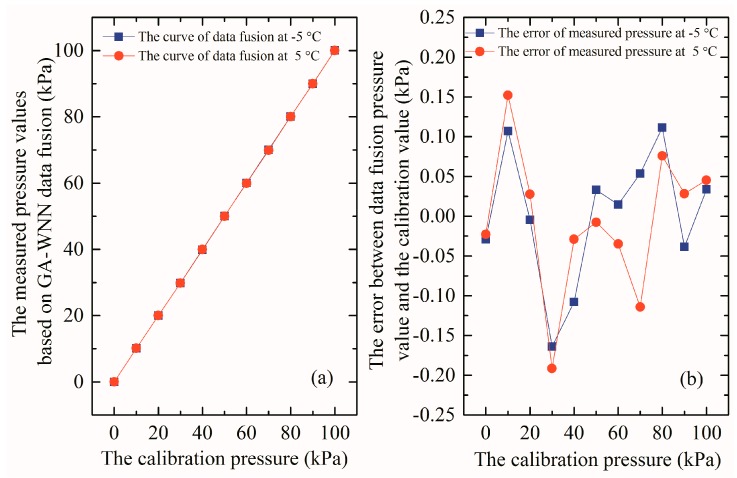
(**a**) The relationship curves between the corrected pressures of array-type sensor and the calibration pressures at different temperature; and (**b**) the absolute error between the corrected pressures of array-type sensor and the calibration pressures at different temperature.

However, we need to further verify this compensation algorithm at actual situation and to ensure reliability under the other temperatures (−5 °C and 5 °C are chosen) [29], and the results are plotted in Figure 18. Figure 18a illustrates the relationship curves between the corrected output pressures of array-type sensor through GA-WNN data fusion and the calibration pressures at different temperatures, which coincides together and are highly linear over the 0–100 kPa range. This result indicates that the influence of the temperature can be neglected once the GA-WNN algorithm is applied. Figure 18b gives the absolute error between the output pressure that is compensated by the GA-WNN algorithm and standard calibration pressure. It can be seen that the absolute error at every calibration pressure point is inconsistent. The reason is as follows: Due to the complexity of calibration sample data, it is very difficult to achieve the same compensation effect at every measurement point by using the GA-WNN algorithm to reduce the measurement error (as shown in Figure 17b,d). In other word, the GA-WNN prediction model can only give the best solution based on the overall compensation effect. Therefore, the phenomenon of inconsistency in the error is normal. Moreover, it can also be found that the maximal prediction error of non-sample data after compensation is 0.20 kPa, which is good because the calibration error in Figure 17b,d is also less than about 0.20 kPa. However, the prediction error is not small enough, which demonstrates that our compensation method still needs to be improved.

**Figure 19:**
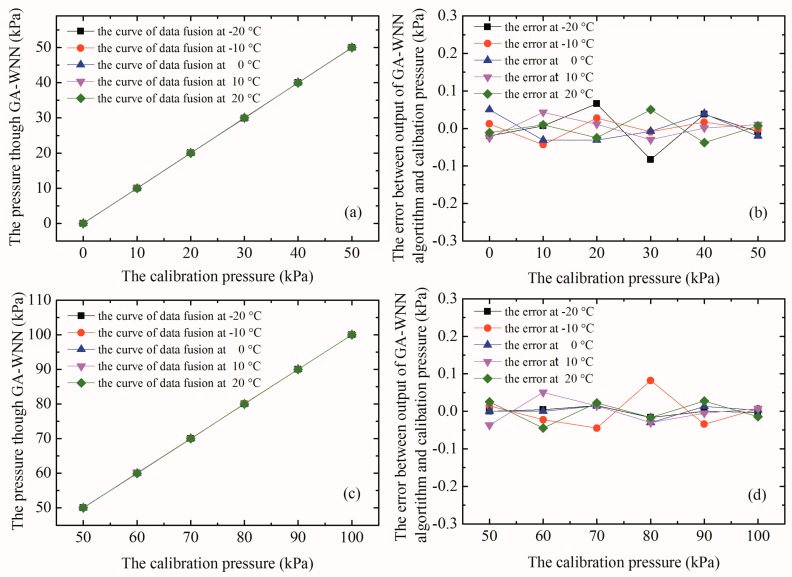
(**a**) The relationship curves between the corrected pressures of PS1/PS2 sensor and the calibration pressures with pressure from 0–50 kPa; (**b**) the absolute error between the corrected pressures of PS1/PS2 sensor and the calibration pressures at different temperature; (**c**) the relationship curves between the corrected pressures of PS3/PS4 sensor and the calibration pressures with pressure from 50–100 kPa; and (**d**) the absolute error between the corrected pressures of PS3/PS4 sensor and the calibration pressures at different temperature.

As mentioned before, the array-type composite sensor is expected to overcome the weaknesses about the low sensitivity of large-range sensor to measure the small pressure and the large nonlinear error of low-range sensor to measure the high pressure. In other words, it can achieve high linearity and sensitivity within the hybrid composite range and meet the accurate measurement in the full range from 0 to 100 kPa. In order to verify the rationality and validity of the basic idea that pressure over the range of 0–50 kPa can be accurately measured by PS1/PS2 sensor and that over the range of 50–100 kPa can be accurately measured by PS3/PS4 sensor, the PS1/PS2 pressure sensor is compensated by GA-WNN in 0–50 kPa range, while the latter is compensated by GA-WNN in 50–100 kPa range. After the optimization algorithm terminates, the results are shown in Table 4 and Figure 19.

It can be seen from Figure 19 that the linearity of the array-type composite pressure sensor has been greatly improved, and the nonlinear errors of the PS1/PS2 and PS3/PS4 pressure sensors calculated from the data in Table 4 drop to 8.330 × 10^−4^ and 8.129 × 10^−4^ in the ranges of 0–50 kPa and 50–100 kPa, respectively. Compared with a single sensor in the 0–100 kPa range, nonlinear error within the hybrid composite range is significantly reduced, which is helpful to improve measurement accuracy of the sensor system. Combined with the data in Table 3, the GA-WNN algorithm (prediction model) is also used to compensate repeatability and hysteresis error. The repeatability error is reduced from 0.158% to 0.152% and the hysteresis error is reduced to 0.183%. It can be seen that GA-WNN prediction model has some compensation effect on the repeatability and hysteresis error of the array-type sensor system. Finally, the overall error of the intelligent pressure sensor system is maintained at ±0.252% within the hybrid composite range (0–50 kPa and 50–100 kPa).

As discussed above, the compensation effect of GA-WNN is good. At this time, connection weights *ω*_1_ between the input layer and hidden layer, connection weights *ω*_2_ between the output layer and hidden layer, scale factor *b*_1_ and shift factor *b*_2_ are:

(13) $$\omega_{1}=\left\{\begin{array}{c} -0.4315,0.8701\\ -0.6180,-0.4040\\ 0.2737,-0.2929\\ 1.0222,-0.4861\\ -0.6555,-0.2931\\ 0.4036,0.1581\\ -0.4187,0.0977\\ 0.1318,0.5307\\ 0.0281,-0.3161\\ -0.0594,-0.3911\\ -0.2884,-0.5976 \end{array}\right\},\;\omega_{2}=\left\{\begin{array}{c} -0.2421\\ 0.3346\\ 0.4708\\ -0.0085\\ -0.2494\\ -0.1281\\ -0.1655\\ -0.7337\\ -0.6527\\ 0.8745\\ 0.6127 \end{array}\right\},\;b_{1}=\left\{\begin{array}{c} 0.0426\\ -0.9849\\ 0.9443\\ -0.3393\\ -0.6601\\ -0.2512\\ -0.5979\\ -0.3923\\ 0.7007\\ -0.2483\\ 1.0740 \end{array}\right\}\text{ and }b_{2}=\left\{\begin{array}{c} 0.1911\\ -0.5695\\ -0.6640\\ -0.1212\\ -0.2582\\ 0.3514\\ 0.5213\\ 1.2292\\ 0.4206\\ 0.7830\\ -0.7024 \end{array}\right\}\text{ for PS1/PS2.}$$

(14) $$\omega_{1}=\left\{\begin{array}{c} -0.5697,-0.3129\\ -0.0767,-0.2895\\ -0.1483,-0.6683\\ 0.0696,0.3103\\ 0.0825,0.5141\\ -0.3235,-0.0784\\ 0.5223,-0.3120\\ -0.4105,0.3803\\ -0.8869,-0.7544\\ 0.1038,0.4993\\ 0.9306,0.0735 \end{array}\right\},\;\omega_{2}=\left\{\begin{array}{c} 0.3292\\ -0.7816\\ -0.7094\\ 1.1221\\ 1.3491\\ -0.6887\\ -0.1572\\ -0.1942\\ 0.1273\\ 0.6728\\ 0.0985 \end{array}\right\},\;b_{1}=\left\{\begin{array}{c} 0.0798\\ 0.4475\\ -0.2523\\ 0.4525\\ 0.3171\\ -0.2435\\ -0.6472\\ -1.0862\\ -0.0224\\ -0.6155\\ 0.4603 \end{array}\right\}\text{ and }b_{2}=\left\{\begin{array}{c} 0.9835\\ -0.9999\\ -0.9823\\ -0.9964\\ -0.9732\\ -0.7078\\ 0.9122\\ -0.4302\\ 0.4231\\ 0.4453\\ 0.2213 \end{array}\right\}\text{ for PS3/PS4.}$$

**Figure 20:**
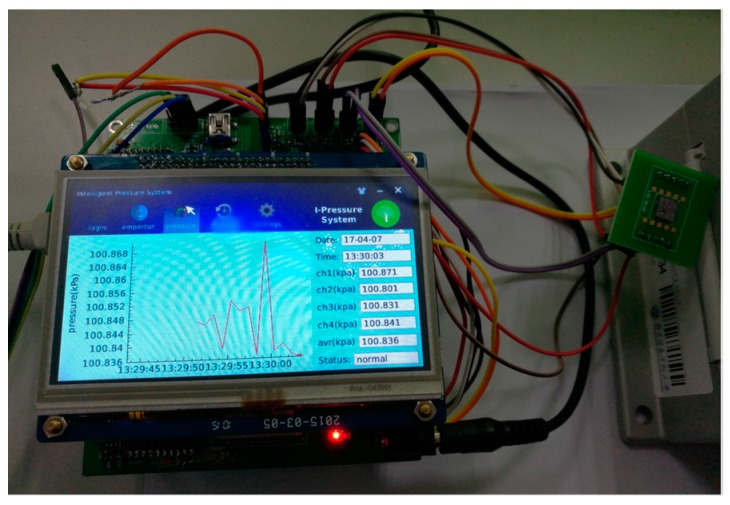
Pressure display interface of piezoresistive MEMS array-type pressure sensor system based on QT and embedded system.

Once the connection weights and other important parameters are obtained, the final weight, threshold, shift factor and scale factor of the trained network are stored as the type of array in the flash memory. According to the GA-WNN compensation algorithm in MATLAB, the GA–WNN data fusion algorithm can be ported to the S3C2440A microprocessor in the form of C language procedures. In this case, the proposed the array-type pressure measuring system can measure and give the actual pressure with temperature compensation in real time. As discussed above, the software implementation process is comprised of four main tasks, and the priority is determined by both the order of the task and its impact on system security. Figure 20 gives the interactive interface of piezoresistive MEMS array-type pressure sensor system, which provides the viewer with the dynamic pressure curve, and letting users simply and enjoyably to utilize it.

## 6. Conclusions and Future Work

In this paper, to effectively solve the problem of the difference in radiosonde measurement accuracy over the entire pressure range (0–100 kPa), we propose a novel idea of using the monolithically integrated array-type composite sensor to measure the pressure in the range of 0–50 kPa and 50–100 kPa, respectively. Specifically, the MEMS array-type piezoresistive pressure sensor employing SOI wafer for radiosonde measurements of pressure is proposed and optimized by ANSYS finite element method. The use of the array enables the designed pressure sensor to have high sensitivity and linearity as well as accuracy. Firstly, the working mechanism of piezoresistive MEMS pressure sensor is analyzed, and the structural and process designs of the array-type pressure sensor are completed. Our studies present a comprehensive analysis of sensor size optimization and doping concentration optimization according to the demands of high sensitivity. We also explore the relationship of piezoresistor size and doping concentration with noise voltage and SNR. The processing and packaging of the array-type pressure sensor chip are realized through the CMOS-MEMS process. Secondly, based on the structural characteristics of the array-type pressure sensor, the functional circuits of data acquisition, digital communication and data storage are designed. The intelligent pressure sensor system is realized based on the S3C2440A microprocessor, and the sensitivity, linearity and temperature drift of the sensor are measured and analyzed. To allow users to utilize the array-type sensor system simply and enjoyably, QT/Embedded graphical interfaces are achieved to display, store and transmit the pressure data. Finally, the temperature and nonlinear as well as hysteresis compensation model of the wavelet neural network based on improved genetic algorithm is proposed. Experimental and test results show that the temperature drift and nonlinear error of the array-type MEMS piezoresistive sensor are well compensated by GA-WNN algorithm, which significantly improves the measurement accuracy of the array-type pressure sensor. Due to the limitations of the manufacturing process, accuracy of the array-type composite pressure sensor we produced is not enough for meteorological measurements at present. However, the current experimental results have shown that when the array-type composite sensor is applied to measure the pressure within two ranges of 0–50 kPa and 50–100 kPa respectively, the difference in measurement accuracy is reduced and the overall measurement accuracy has improved. Of course, this improvement is not yet fully demonstrated. If one expands the dynamic range of the pressure measurement, the efficacy of “multi-range” detection of pressure will be sufficiently shown. In future, for other applications such as downhole pressure sensor, we will expand the design method of the array-type pressure sensor to a larger dynamic range.

## Figures and Tables

**Table 1:** The calibration sample data of PS1/PS2 sensors for the training of the GA-WNN, which are obtained with pressures from 0 to 100 kPa and with temperatures from −20 to 20 °C.

Temperature	*P*/kPa	0	10	20	30	40	50	60	70	80	90	100
*T* = −20 °C	*U_p_*/mV	1.506	4.804	8.128	11.433	14.745	18.036	21.348	24.670	27.956	31.265	34.597
*T* = −10 °C	*U_p_*/mV	1.295	4.428	7.596	10.738	13.846	16.997	20.134	23.291	26.298	29.523	32.696
*T* = 0 °C	*U_p_*/mV	0.865	3.942	7.062	10.043	13.191	16.292	19.434	22.465	25.570	28.638	31.858
*T* = 10 °C	*U_p_*/mV	0.564	3.632	6.732	9.790	12.862	15.943	19.037	22.126	25.179	28.285	31.276
*T* = 20 °C	*U_p_*/mV	0.052	2.936	5.832	8.748	11.625	14.542	17.421	20.336	23.242	26.104	29.020

**Table 2:** The calibration sample data of PS3/PS4 sensors for the training of the GA-WNN, which are obtained with pressures from 0 to 100 kPa and with temperatures from −20 to 20 °C.

Temperature	*P*/kPa	0	10	20	30	40	50	60	70	80	90	100
*T* = −20 °C	*U_p_*/mV	−0.010	1.270	2.554	3.856	5.141	6.416	7.708	8.990	10.268	11.563	12.846
*T* = −10 °C	*U_p_*/mV	−0.050	1.211	2.474	3.765	5.031	6.288	7.568	8.830	10.103	11.377	12.638
*T* = 0 °C	*U_p_*/mV	0.007	1.182	2.394	3.606	4.811	5.994	7.168	8.370	9.571	10.804	12.002
*T* = 10 °C	*U_p_*/mV	0.047	1.178	2.342	3.542	4.698	5.858	7.010	8.152	9.308	10.498	11.638
*T* = 20 °C	*U_p_*/mV	−0.034	1.122	2.230	3.404	4.540	5.684	6.794	7.933	9.064	10.240	11.357

**Table 3:** The output voltages of the array-type sensor without compensation during the pressure increasing and pressure decreasing cycle at 20 °C.

*P*/kPa	10	20	30	40	50	60	70	80	90	100
*U*_inc_/mV	1.123	2.229	3.401	4.538	5.669	6.795	7.933	9.064	10.222	11.342
*U*_dec_/mV	1.140	2.246	3.422	4.556	5.692	6.811	7.954	9.080	10.231	11.346
*U*_inc_/mV	1.132	2.222	3.386	4.541	5.675	6.792	7.933	9.065	10.228	11.357
*U*_dec_/mV	1.141	2.240	3.405	4.558	5.694	6.812	7.949	9.082	10.239	11.357
*U*_inc_/mV	1.122	2.230	3.404	4.540	5.684	6.794	7.933	9.064	10.240	11.356
*U*_dec_/mV	1.138	2.247	3.421	4.563	5.705	6.812	7.951	9.082	10.241	11.357
*U*_inc_/mV	1.125	2.230	3.402	4.539	5.680	6.794	7.934	9.064	10.233	11.351
*U*_dec_/mV	1.140	2.246	3.422	4.561	5.701	6.811	7.952	9.081	10.236	11.353

**Table 4:** The predicted output *P_GW_* of array-type pressure sensor though GA-WNN algorithm within the hybrid composite range.

Temperature	*P*/kPa	0	10	20	30	40	50	60	70	80	90	100
*T* = −20 °C	*P_GW_*/kPa	−0.02	10.01	20.07	29.92	40.04	49.99	60.00	70.02	79.98	90.00	100.00
*T* = −10 °C	*P_GW_*/kPa	0.01	9.96	20.03	29.99	40.02	50.00	59.98	69.96	80.08	89.97	100.01
*T* = 0 °C	*P_GW_*/kPa	0.05	9.97	19.97	29.99	40.04	49.98	60.00	70.01	79.97	90.01	100.00
*T* = 10 °C	*P_GW_*/kPa	−0.02	10.04	20.01	29.97	40.00	50.01	60.05	70.01	79.97	89.99	100.01
*T* = 20 °C	*P_GW_*/kPa	−0.01	10.01	19.98	30.05	39.96	50.01	59.96	70.02	79.98	90.03	99.99
